# Shrimp thrombospondin (TSP): presence of *O*-β1,4 N-acetylglucosamine polymers and its function in TSP chain association in egg extracellular matrix

**DOI:** 10.1038/s41598-022-11873-7

**Published:** 2022-05-13

**Authors:** Sirilug Magerd, Thanyaporn Senarai, Orawan Thongsum, Chidchanok Chawiwithaya, Chihiro Sato, Ken Kitajima, Wattana Weerachatyanukul, Somluk Asuvapongpatana, Piyaporn Surinlert

**Affiliations:** 1 Department of Basic Medical Science, Faculty of Medicine Vajira Hospital, Navamindradhiraj University, Bangkok, Thailand; 2 Department of Anatomy, Faculty of Medicine, Khon Kaen University, Khon Kaen, Thailand; 3 Department of Anatomy, Faculty of Science, Mahidol University, Bangkok, Thailand; 4 Bioscience and Biotechnology Center and Graduate School of Bioagricultural Sciences, Nagoya University, Nagoya, Japan; 5 Chulabhorn International College of Medicine, Thammasat University, Pathum-Thani, Thailand; 6 Research Unit in Synthesis and Applications of Graphene, Thammasat University, Pathum-Thani, Thailand

**Keywords:** Cell biology, Molecular biology

## Abstract

We characterized the existence of *O*-β(1,4)-GlcNAc polymers (β1,4GNP) that were anchored on the *O*-linked glycosylation sites of shrimp thrombospondin (*pm*TSP-II). There were five putative β1,4GNP linkages on the epithelial growth factor-like domain of *pm*TSP-II. Antibody against *O*-β-GlcNAc (CTD110.6) was used to prove the existence of linear and complex β1,4GNP. The antibody well reacted with linear chito-triose, -tetraose and -pentaose conjugated with phosphatidylethanolamine lipid. The immunoreactivity could also be detected with a complex β1,4GNP within *pm*TSP-II (at MW > 250 kDa). Upon denaturing the protein with SDS-PAGE buffer, the size of *pm*TSP-II was shifted to be 250 kDa, approximately 2.5 folds larger than the deduced molecular mass of *pm*TSP-II (110 kDa), suggesting additional association of *pm*TSP-II apart from its known disulfide bridging. This was confirmed by chitinase digestion on *pm*TSP-II protein leading to the subsequent smaller protein bands at 110–170 kDa in time- and concentration-dependent manners. These bands well reacted with CTD110.6 antibody and disappeared after extensive chitinase hydrolysis. Together, we believe that β1,4GNP on *pm*TSP-II serve the function in an inter-chain association to provide structural architecture of egg extracellular matrix, a novel function of *pm*TSP-II in reproductive biology.

## Introduction

Despite being composed of only three or four glycoproteins, the multiple roles of egg vestments, such as zona pellucida (ZP) in mammals or ZP-related egg coats in other animal species are well known during gamete fertilization processes. Apart from the peptide chains, their glycoproteins are known to be heavily glycosylated with both *N*- and *O*-linked glycoconjugates which can make up > 30–50% of their total molecular masses^1,2^. Through high throughput technology, the extensive genomic and proteomic information of the nascent ZP peptides or ZP-related glycoproteins have been well characterized and extensively deposited in many available databases^3,4^. Information about carbohydrate moieties on the ZP glycoproteins, on the other hand, is limited, even in the most extensively studied mouse model, due to the fact that the complex structure of carbohydrates (both their length and branching patterns) are non-uniform and therefore difficult to predict its molecular structures. As a consequence, the role of each individual carbohydrate species on the same egg glycoprotein (such as ZP3) appears highly heterogenous and is still a debatable issue among researchers. One of the most contentious issues is whether the *N*-linked or *O*-linked glycans (or both) on ZP3 participate in gamete interaction leading to acrosome induction^5–7^. Apart from being important receptors and inducers during fertilization processes, recently research has suggested that carbohydrate moieties play a non-fertilization role in regulating the structure of the mammalian egg ZP. It has been reported that all ZP glycoproteins interact both covalently and noncovalently among their filaments to form the helical-like bundle of extracellular matrix^8^. Nonetheless, detailed study of the types of carbohydrate species that are involved in the formation of this helical-like structure is still incomplete.

Shrimp ovulated oocytes are surrounded by a jelly layer which is derived from the peripherally embedded cortical rod (CR) materials just prior to sperm interaction^9^. It has long been known that cortical rod components are heavily glycosylated, having about 70–75% carbohydrate constituents^10,11^. After egg spawning, the extruded CRs on the egg surface become a flocculent material known as egg water (EW) which is inherently an inducer of sperm acrosome reaction^12^. Among many CR proteins, thrombospondin protein (TSP) has been well-characterized in two separate shrimp species, termed TSP-I in *F. chinensis*^13–15^ and *pm*TSP-II in *Penaeus monodon*^16,17^. As a multi-domain protein, it has been suggested that TSP has a broad range of physiological functions, including polyspermy barrier, anti-bacterial agent, ovarian development and inducer of acrosome reaction^13,14^. Recently, the novel function of the *N*-linked mannosyl glycoconjugates on the calcium binding domain (TSP3 domain) of *pm*TSP-II has been shown to be crucial for sperm AR induction^17^. This finding suggests the role of carbohydrates in mediating the fertilization step of shrimp, in a similar manner to that reported in mammals, mentioned above. In this study, we extrapolated our findings to include *O*-linked glycoconjugates, β1,4GNP in the EGF-like domain of *pm*TSP-II and unraveled its role in TSP-interchain association, a novel non-fertilization role in shrimp gamete biology.

## Results

### The potential *O*-β-GlcNAc glycosylation sites on EGF-like domain of *pm*TSP-II

**Figure 1:**
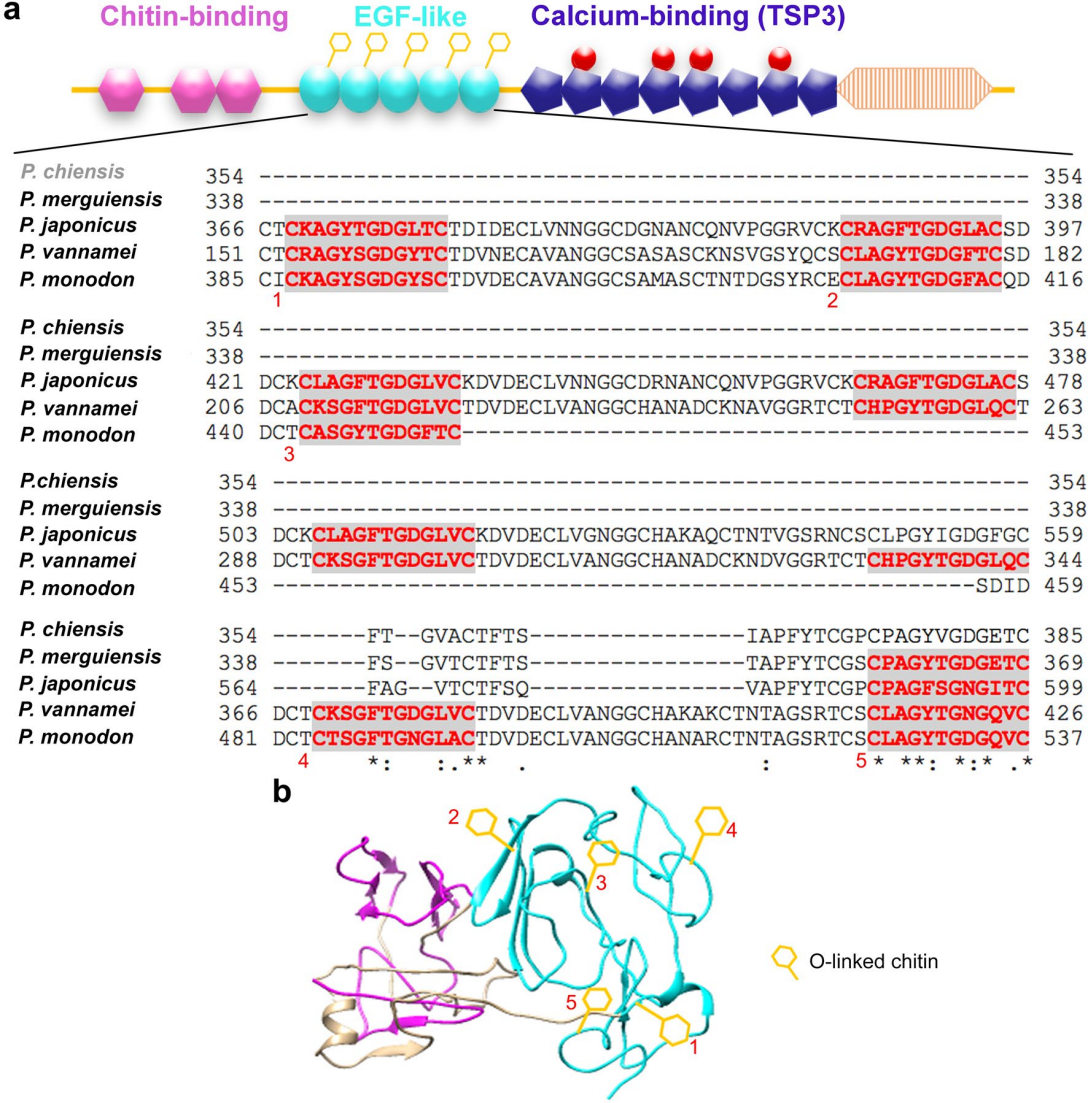
The potential sites of *O*-β-GlcNAc modification on penaeid shrimp species including *P. chiensis*, *P. merguiensis*, *P. japonicus*, *P. vannamei* and *P. monodon*. Alignment of the EGF-like domain in *pm*TSP-II was performed by Clustal omega program (**a**). The whole structure of *pm*TSP-II was shown in the two-dimensional structure, which composed is of chitin binding domain, EGF-like domain and calcium binding domain. The *O*-β glycosylated motifs were in red bold text with gray shading. The three-dimensional structure of *pm*TSP-II with *O*-linked projecting on EGF-like module were predicted and created by PyMOL program (**b**). The five motifs of *O*-β-GlcNAc (yellow) are presented on the EGF-like domain (blue color).

Our previous study reported that the full sequences of *pm*TSP-II comprised three important signature domains: chitin binding domain (CBD), EGF-like domain, and calcium-binding domain or TSP3 domains^16^. Here, we searched for the potential sites of *O*-β-GlcNAc glycosylation based on the CXXG(Y/F)(T/S)GZ_2-5_C amino acid sequence motif that was conserved throughout TSP’s protein family^18^. This sequence could be found in many penaeid shrimp species including *P. monodon*, *P. merguiensis*, *P. vannamei* and *P. japonicus* (Fig. 1a). Interestingly, the *O*-β-GlcNAc glycosylation sites were found exclusively within the middle part of the EGF-like domain. In *P. monodon*, up to five *O*-β-GlcNAc anchoring sites were found. These numbers, however, varied from one (in *P. merguiensis*), to six (*P. japonicus*) and up to the maximum of eight potential sites in *P. vannamei* (red bold capital letters with gray shading). The site was, however, missing in *P. chinensis,* even though the TSP sequence in this species is closely related to other penaeid shrimp species^16^. In the two-dimensional linear arrangement of multi-domains in *pm*TSP-II, the five positions of *O*-β-GlcNAc were evenly distributed within the EGF-like domain (Fig. 1a, blue box). The deduced three-dimensional structure of *pm*TSP-II suggested that the β1,4GNP exposed outward from the surface of *pm*TSP-II molecule (Fig. 1b). We thus believe that this arrangement of *pm*TSP-II would favor the interaction between β1,4GNP and CBD in the adjacent *pm*TSP-II peptide chain to form the supramolecular architecture of *pm*TSP-II.

### Detection of O-β-GlcNAc residues on the conjugated lipid and the isolated CR proteins

**Figure 2:**
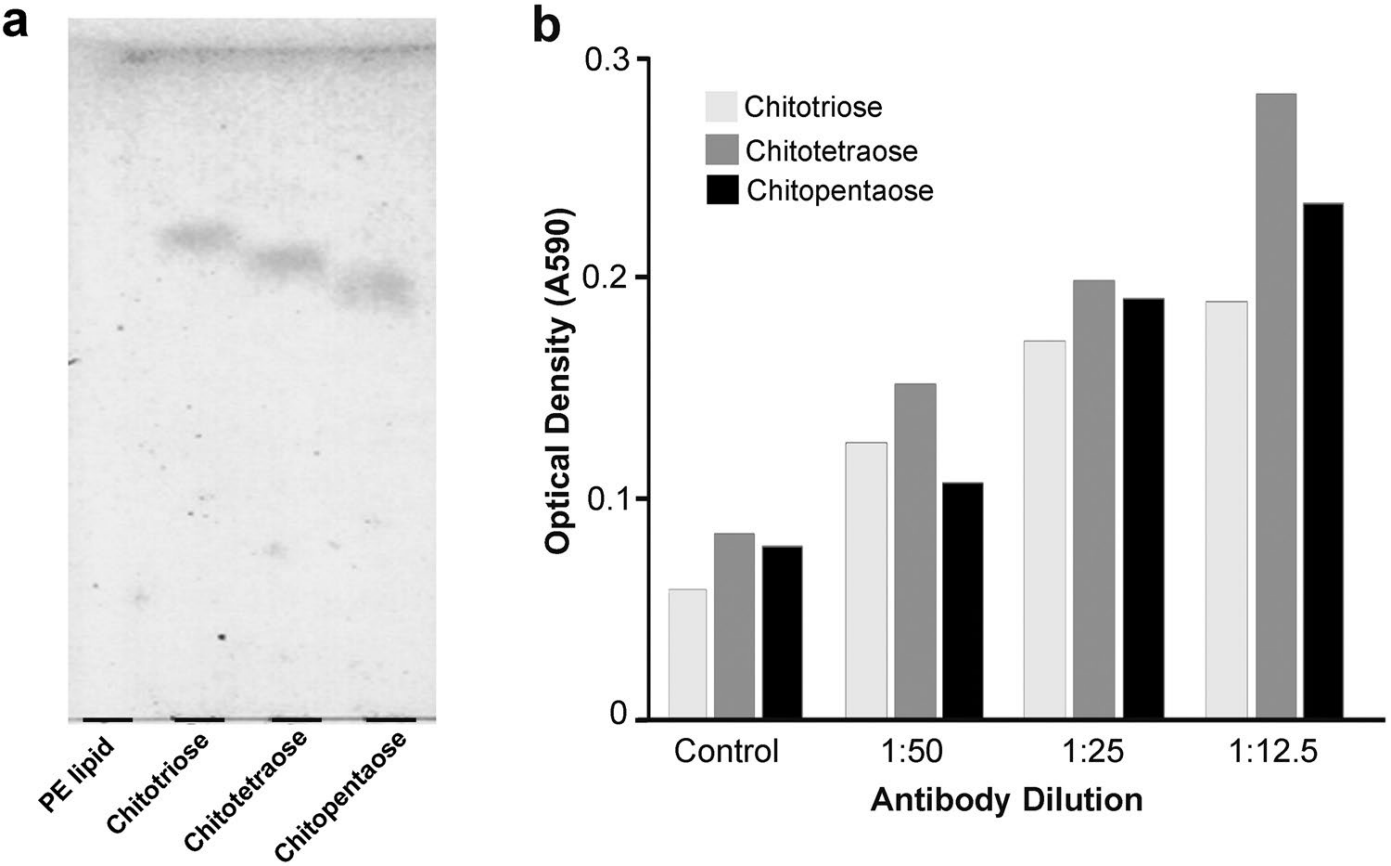
The affinity of a CTD110.6 antibody with a linear *O*-β-GlcNAc-linked phosphatidylethanolamine (PE) and its quantitative analysis. A linear *O*-β-GlcNAc chain including chitotriose, chitotetraose and chitopentaose conjugated with PE lipid were analyzed by TLC plate and visualized by orcinol staining (**a**). Quantitative binding of a CTD110.6 antibody towards *O*-β-GlcNAc-linked PE was performed by ELISA assay in the various concentrations (**b**).

As the deduced amino acid sequence of *pm*TSP-II indicated the anchorage of *O*-β-GlcNAc residues, we therefore aimed to investigate the existence of the complex *O*-β-GlcNAc chitin chain in the purified *pm*TSP-II using a CTD110.6 antibody. Since the antibody has been proven to recognize *O*-β-GlcNAc monosaccharide^19^, its binding with the oligomeric *O*-β-GlcNAc linked to lipid substrate was initially tested. The thin layer chromatograph in Fig. 2a clearly demonstrated a successful linkage of *N*-acetyl-triose, -tetraose and -pentaose (as a purplish-blue enzyme precipitate resulting from the charring reaction of carbohydrates with an orcinol) to the PE lipid. The longer the sugars attached to the PE lipids, the slower the band mobility observed. Through an ELISA analysis, these oligomeric sugar-lipid linkages were well recognized by a CTD110.6 antibody with a dilution factor ranging from 1:12.5 to 1:50 and optical density from 0.05 to 0.3 (Fig. 2b). This result extrapolated the recognition of this antibody from monomeric sugar towards linear chained sugars.

### Localization of O-β-GlcNAc modification on the cortical rods of mature shrimp ovaries

**Figure 3:**
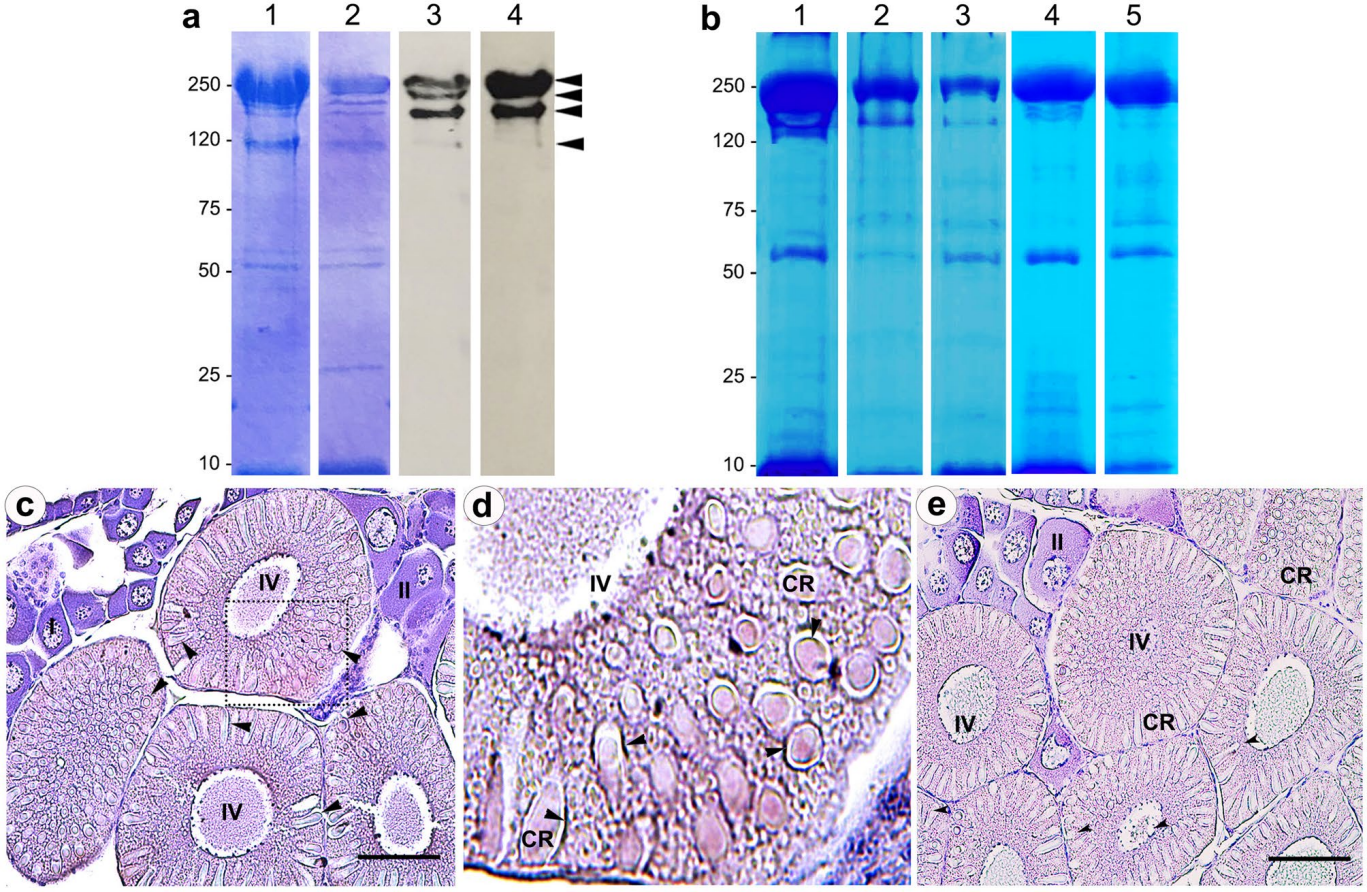
Existence of *O*-β-GlcNAc polymers in TSP-like protein in the cortical rods (CRs) of mature oocytes. The extracted soluble CR proteins (without protease inhibitor) at the different storage periods were subjected to SDS-PAGE and Coomassie blue staining (panel **a**, lanes 1 and 2) or probing either with CTD110.6 or anti-TSP antibody (lanes 3 and 4, respectively). Note the similar staining patterns of both a CTD110.6 and anti-TSP antibodies. Freshly purified wsCR (panel **b**, lane 1) was treated with deglycosylation agents, TFMSA and NaIO_4_ (panel **b**, lanes 2 and 3, respectively) and with denaturizing agents, 7 M Urea in 0.1 N HCl (lane 4) and 6 × loading dye (lane 5) followed by staining with Coomassie blue. Panel (**c**) represents localization of *O*-β-GlcNAc polymers in mature shrimp ovary stained by CTD110.6 antibody revealing an intense staining as dotted-like granules throughout cytoplasm and around the periphery of CRs (arrowheads). I, II, IV are oocyte stages I, II and IV, respectively. The enlarged picture of dashed-line box is shown in (**d**), while negative control is shown in (**e**). Bars = 200 µm.

It has been shown that the deduced molecular mass of a nascent peptide of *pm*TSP-II (without glycosylation) is approximately 110 kDa^16^. Upon its fresh purification by FPLC and dissolving by SDS-PAGE, the major band of 250 kDa was achieved (Fig. 3a, lane 1). However, after leaving the protein in suspension for a few weeks without protease inhibitors, the 250 kDa band was gradually hydrolyzed into the serially smaller bands at approximately 170, 150 and 110 kDa (Fig. 3a, lane 2). These proteins intensively reacted with anti-TSP (Fig. 3a, lane 4), suggesting that they are the multimeric forms existing in the extracellular matrix of the eggs. Using the CTD110.6 antibody to probe the purified *pm*TSP-II, similar immunoreactive bands as those of anti-TSP namely, 250 kDa, 170 kDa, 150 kDa and 110 kDa were revealed (Fig. 3a, lane 3, arrow heads). Interestingly, incubation of purified proteins with deglycosylation agents (trifluoromethanesulfonic (TFMSA) and sodium metaperidate (NaIO_4_) resulted in a decreased intensity of the 250 kDa band and the appearance of 150 kDa and the other smaller banded proteins (Fig. 3b, lanes 2 and 3). In addition,exposure of the purified 250-kDa protein to strong denaturing agents did not grossly affect the intensity of the 250 kDa band, suggesting that the structural organization of the 250 kDa *pm*TSP-II is rather dependent on interaction of carbohydrate than disulfide bridges or hydrogen bonding.

We further performed the localization of β1,4GNP in vivo using paraffin sections of the mature shrimp ovary. Strong immunoreactivity of CTD110.6 antibody was detected on the peripheral rim of the CRs in the stage IV oocyte (Fig. 3c,d, arrowheads). A moderate staining could also be seen in the cytoplasmic granules of the stages II and III oocytes. Negative control revealed minimal antibody staining in the stage IV oocyte (Fig. 3e).

### β1,4GNP of pmTSP-II is involved in the supramolecular chain-association

**Figure 4:**
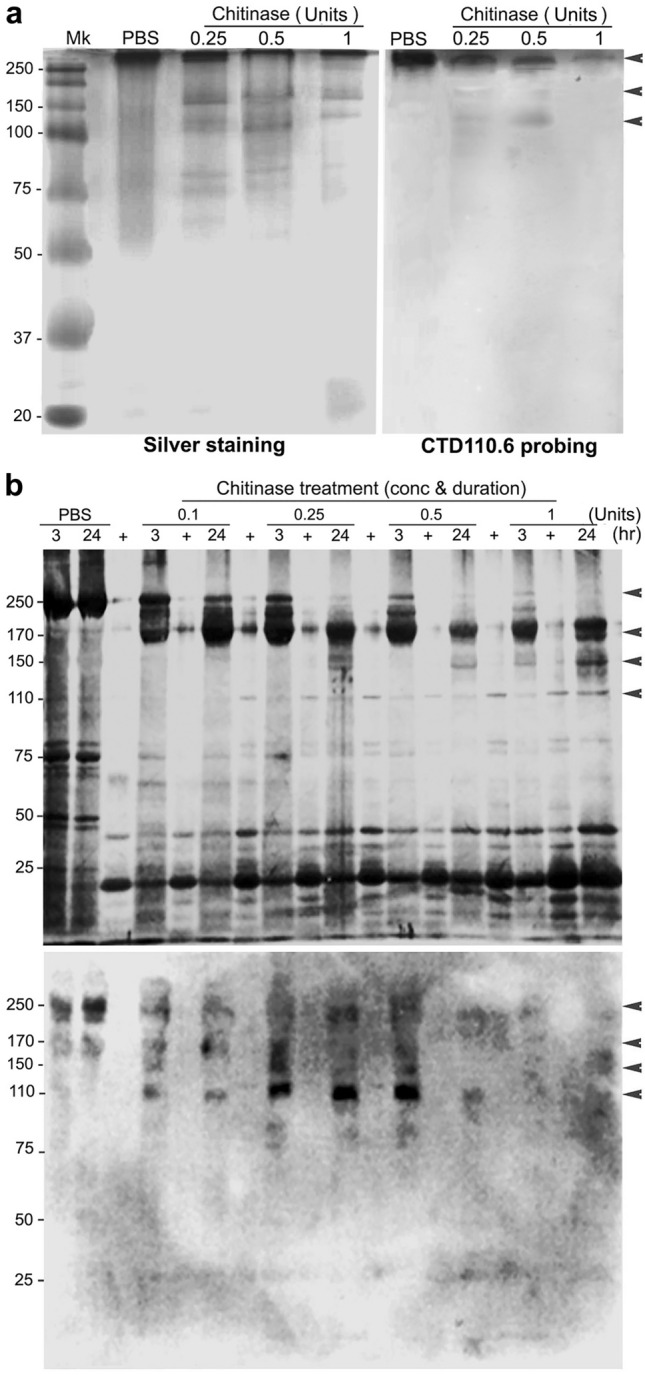
Native and denatured protein profiles of the chromatographically purified *pm*TSP-II upon subjecting to chitinase digestion and their probing with a CTD110.6 antibody. The proteins were treated with the different units of chitinase and were resolved by native gel electrophoresis followed by silver staining (**a**, left panel) or probed with a CTD110.6 antibody (right panel). The purified *pm*TSP-II is shown in PBS lane and followed by the treatment with 0.25–1 unit chitinase. Panel (**b**): a denaturing SDS-PAGE condition of the similarly chitinase-treated *pm*TSP-II followed by silver staining (above) or CTD110.6 antibody probing (below). The reactivity of purified *pm*TSP-II that was subjected to chitinase digestion at the various concentrations (0.1–1 units) and time intervals (**b**, above). + indicates chitinase loading and arrowheads indicate CTC110.6 antibody-reactive bands corresponding to its mobility shown in silver staining.

Despite many reports indicating the presence of cysteine-based disulfide bridging that is involved in a supra-molecular structure of mammalian TSP proteins (whose deduced molecular mass is about 110–120 kDa)^20^, our native gel electrophoresis showed the clustered of *pm*TSP-II protein at the molecular mass much higher than 250 kDa (Fig. 4a, left panel, lane 2) which was intensely reactive with CTD110.6 antibody (right panel, arrowhead). Under the denaturing condition of SDS-PAGE, the results demonstrated a still-sizable 250 kDa *pm*TSP-II, even though they were exposed to a strong reducing agent and detergent in the PAGE resolving buffer (Fig. 4b, upper panel, lane PBS). We therefore aimed to search for the supramolecular cross-linkage of *pm*TSP-II strands, one of which could be a carbohydrate binding with its complimentary peptide domain, CBD. A bioinformatics analysis of *pm*TSP-II sequence, contained both an *O*-β-GlcNAc anchorage site on the *N*-terminus EGF-like domain as well as several CBD in the middle part of the molecule (Fig. 1a), a unique feature of many reported crustacean TSPs. Cross-interaction between β1,4GNP and CBD could thus, hypothetically, generate a multimeric-bundling structure of *pm*TSP-II.

We tested this hypothesis by a chitinase digestion of the β1,4GNP on the freshly purified *pm*TSP-II (mainly 250 kDa) which should have dissolved its bundling structure, resulting in a singlet chain of a 110 kDa protein. In the native condition, *pm*TSP-II that was treated with 0.25–1 U chitinase (24 h) showed the gradually reduced molecular mass of *pm*TSP-II to be the smaller sized proteins, particularly 110 and 150 kDa bands (arrowheads) and some other smaller bands (Fig. 4a, left panel, lanes 3–5). Reactivity of these digested proteins with a CTD110.6 antibody was also detected at 250 kDa band (in a decreased intensity manner upon increasing chitinase concentrations) and as a faint to moderate intensity at 150 and 110 kDa bands (Fig. 4a, right panel, lanes 2–4). In denaturing SDS-PAGE (Fig. 4b), it was apparent that the molecular mass of *pm*TSP-II gradually reduced to the smaller bands of 110–170 kDa bands (Fig. 4b, upper panel). Either longer exposure to 0.25 U chitinase (3–24 h) or higher amounts of enzyme (0.5–1 U) led to an apparent reduction of the upper bands (250 and 170 kDa) while increasing the intensity of the 150 and 110 kDa bands (lanes 12–18). Moreover, when the purified *pm*TSP-II proteins were probed with a CTD110.6 antibody, the 250 kDa non-digested protein was intensely reactive with the antibody (Fig. 4b, lower panel, lanes 1–2). Reactivity of CTD110.6 antibody gradually shifted towards the lower molecular weighted bands, particularly the 110 kDa protein, in the 0.1–0.25 U chitinase-digested proteins (lanes 8–10). The prolonged or intensely digested samples with 0.5–1 U chitinase (lanes 12–18) showed very low or complete absence of antibody reactivity, suggesting a complete removal of β1,4GNP from the *pm*TSP-II peptide core. The results thus indicated the significance of β1,4GNP within the EGF-like domain in the supramolecular association of *pm*TSP-II within the egg extracellular matrix.

## Discussion

The significance of carbohydrate moieties in egg vestments, either *N*-linked or *O*-linked glycoconjugates has become unraveled in the fertilization process, both in mammals and invertebrates^12,21,22^. However, there is less evidence demonstrating the structural role of specific carbohydrate moieties, even in model animals, such as mice and sea urchins. Among mouse ZP glycoproteins, some suggestions have been made about the involvement of carbohydrates in ZP structural formation^6,8^. Experimental evidence suggested that molecular bundling of ZP filaments can be dissolved in various conditions such as mild acid, heat and strong reducing agents, suggesting the involvement of both disulfide bridging and other non-covalent bonding such as carbohydrate-carbohydrate interaction^6,7,23^. If this is the case, the specific carbohydrate types involved in this interaction need to be explored. In marine invertebrates, we believe that structural organization of the egg vestments is more dependent on carbohydrate-carbohydrate or carbohydrate-protein interaction. This is simply due to their structural appearance which is more gelatinous^24,25^ compared with the dense filamentous nature of mammals. Here, we have shown evidence of the involvement of the *O*-β-GlcNAc chitin chain in the association of *pm*TSP-II bundling. Bioinformatics analysis indicated the presence of an *O*-linked glycosylation motif, CXXG(Y/F)(T/S)GZ_2-5_C exclusively in the EGF-like domain of *pm*TSP-II and TSPs of other shrimp species (Fig. 1) similar to that in an insect species, *D. malagaster*^26^. The number of O-linked glycosylation sites vary among penaeid shrimp species where *P. monodon, P. vannamei and P. japonicus* contain 5–8 *O*-β-GlcNAc modification sites, whereas *P. merguensis* has only one site. It is noted in one penaeid shrimp species, *P. chiensis,* that even with the presence of EGF-like domain, but it still lacks an *O*-β-GlcNAc glycosylation site (Fig. 1a), an interesting evolutionary difference that remains to be addressed.

Generally, protein polymerization to form the supramolecular structure through the disulfide bridges has been a priority. This also holds true in the case of mammalian TSPs that have previously been documented^27,28^. In such cases, deterioration of disulfide bridging with strong reducing agents greatly affects protein structure and produces TSP chain singlets^29^. Among shrimp TSPs studied, their analyzed molecular constituents also comprised many putative cysteines that potentially form the disulfide bonds needed for higher molecular hierarchical formation^13–16^. This is also the case of *pm*TSP-II where its deduced amino acid sequences are composed of all signature domains of TSPs as well as many putative cysteine residues. However, the fact that exposing *pm*TSP-II to the reducing agents did not affect a 250-kDa polymer of *pm*TSP-II, but rather treating it with chitinase enzyme would favor the chitin-based polymerization of *pm*TSP-II (Fig. 4). Therefore, the presence of several chitin binding domains (CBD) in shrimp TSPs would thus be an alternative bonding for clustering adjacent TSP chains to form bundles of TSP polymers. In fact, the role of β1,4GNP in molecular bundling has been well documented for the formation of many crustacean shells through a side-by-side stacking of chitins which are held together by hydrogen bonding^30,31^. This ends up with a super-large molecular mass of chitin polymers in several kinds of chitin-based shells or cuticles. Alternatively, the other bonding type of chitin chains with their complimentary sites on the nascent peptides (known as CBD) has also been mentioned in some literature^32^ but not for shrimp TSPs. This latter bonding type of β1,4 GNP or chitin would be more favorable for *pm*TSP-II because of the existence of a number of CBD in the entire crustacean TSP molecule. Bundling of TSP chains through β1,4GNP-CBD interaction would also help to explain why shrimp TSPs engage many CBDs in their molecular structures which are rather unique to these animal species.

**Figure 5:**
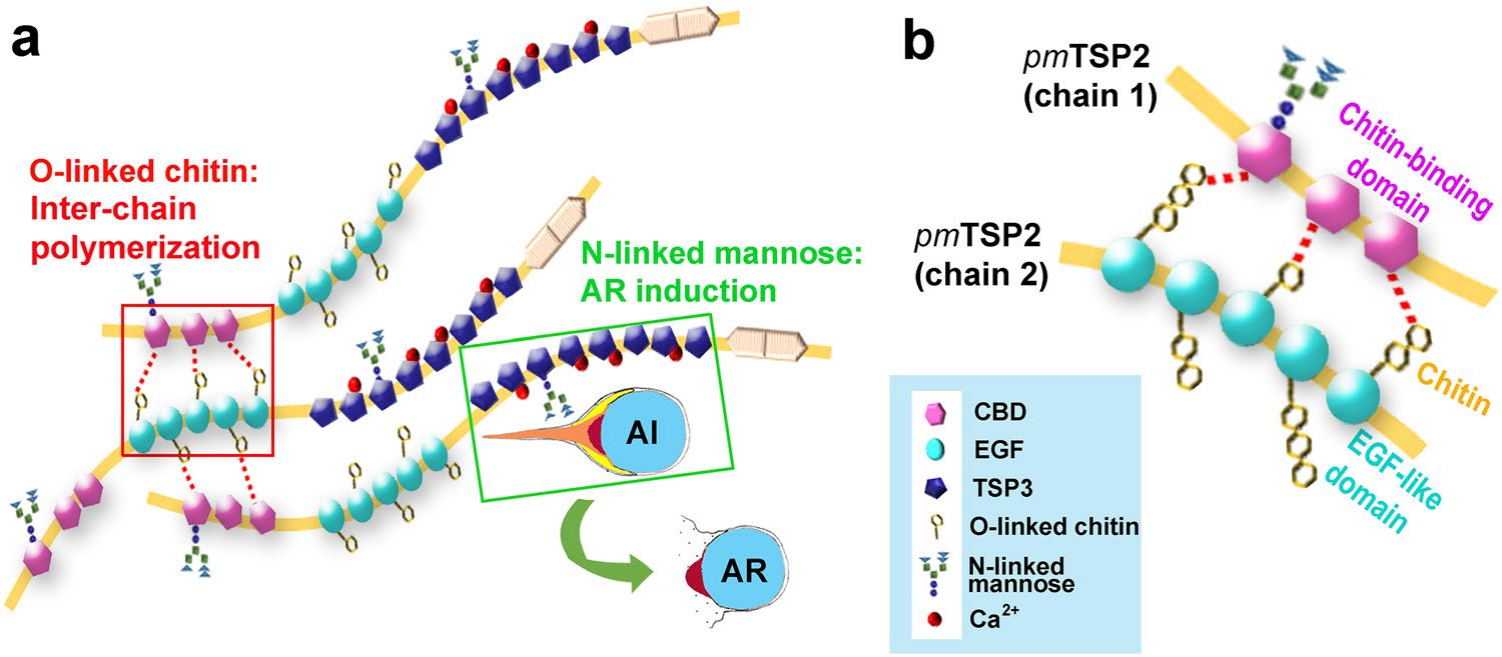
A schematic drawing by Photoshop (CS5 program software) demonstrating the significance of *pm*TSP-II’s carbohydrate moieties in shrimp reproductive biology. (**a**) *O*-β-GlcNAc polymers anchored in the EGF-like domain (blue spheres) interact with CBDs (pink hexagons) of the adjacent *pm*TSP-II chains to form a supramolecular structure of *pm*TSP-II. *N*-linked mannosyl glycoconjugates on CBD or TSP3 (purple pentagons) interact with not-yet characterized the receptor on the acrosome intact (AI) sperm (green boxed), which led to the subsequent rupture of the acrosomal sac as part of the sperm acrosome reaction. (**b**) The proposed detailed interaction between *O*-β-GlcNAc polymers and CBD through carbohydrate-protein interaction (red dashed lines).

Shrimp TSPs have been known to serve several functions, including cellular immune responses^33–35^ or fighting against bacterial infection^13,14^. It is still unclear whether these physiological functions would have been derived from their peptide core or carbohydrate moieties. In the case of *P. monodon*, the significance of both *N*- and *O*-linked glycoconjugates in *pm*TSP-II was evident and summarized in Fig. 5. The *N*-linked mannose glycoconjugates in CBD and TSP3 domains have been shown to be involved in AR induction^17^. β1,4GNP presumably anchored in the EGF-like domain was characterized by its reactivity with CTD110.6 antibody (Figs. 3 and 4) and may favorably interact with CBD of the adjacent *pm*TSP-II chains to form the supramolecular structure (Fig. 5a,b red dashed line). Perspectively, knocking down of *pm*TSP-II gene either during oocyte development or their spawning at shrimp spent phase through RNAi technology should give a further insight about the function of *pm*TSP-II in the structural organization of the egg extracellular matrix (or cortical rods). All together, our results reveal the novel significance of carbohydrate moieties on *pm*TSP-II, both in fertilization and non-fertilization functions in marine shrimp species.

## Materials and methods

### Molecular analysis of pmTSP-II and its carbohydrate anchoring sites

Amino acid sequences of thrombospondin (TSP) in *P. monodon* (AGI56293.2), *F. chinesis* (AAZ66372.1), *F. merguiensis* (ACV32380.1), *P. japonicus* (BAC92762.1) and *P. vannamei* (ROT68707.1) were obtained from the GenBank database. The sequences were analyzed by multiple sequence alignments using a Clustal Omerga program (http://www.ebi.ac.uk). Comparison of the predicted domains, repeats, motifs and features of TSPs in different animals were performed with an InterProScan software (http://www.ebi.ac.uk/Tools/pfa/iprscan/). The consensus *O*-linked *N*-acetylglucosamine (*O*-β-GlcNAc) on TSP (CXXG(Y/F)(T/S)GZ_2-5_C) motif was also analyzed. The 3D structure was deduced from a known sequence of *pm*TSP-II using the Phyre2 server program (http://www.sbg.bio.ic.ac.uk/phyre2/html/page.cgi?id=index)^36^ and analyzed by a PyMOL software (The PyMOL Molecular Graphics system, Schrodinger, LLC).

### Isolation of cortical rods (CRs) and purification of pmTSP-II

Wild-caught fully mature female *P. monodon* weighing 400–500 g captured from the Gulf of Thailand were obtained from a commercial farm in Chachoengsao province (Thailand). They were acclimatized in 500-L plastic laboratory tanks at the ambient temperature (28 °C) with seawater at the salinity of 20 ppt at least one day before they were used in the experiments. Shrimps were handled according to the guidelines of the Animal Care Committee, Mahidol University (MUSC-IACUC, protocol # 2016/014). Briefly, they were anesthetized by placing in ice for 5 min and dissected carefully to collect the ovaries. The pieces of mature ovary were fixed in Davidson fixative (Sigma-Aldrich, St. Louis, MO) and processes for immunohistochemistry or kept frozen for CR isolation.

Isolation of CRs was performed according to previously described protocol^12^. Briefly, mature ovary was homogenized in an isolation medium (IM: 500 mM NaCl, 9 mM CaCl_2_, 14 mM KCl, 15 mM MgCl_2_, and 10 mM Tris, pH 7.6) containing 30% sucrose. Thereafter, the suspension was centrifuged at 1000 × *g* for 5 min to harvest the pellet, which was then resuspended in IM and overlaid in 40% sucrose, followed by centrifugation (8000 × *g*, 4 °C, 60 min). The yolk contaminants were washed away from CRs by single step centrifugation (1000 × *g*, 4 °C, 5 min) through a 30% sucrose in IM. The CR was then subjected to protein extraction in lysis buffer with and without protease inhibitor (1 mM PMSF + protease inhibitor cocktails (Sigma-Aldrich, St. Louis, MO). Protein concentration was determined by a BCA assay kit (Thermo Fisher Scientific, Waltham, MA). Purification of *pm*TSP-II protein was then performed as described^17^ through Sepharose G-300 size-exclusion chromatography, (Amersham Pharmacia, Uppsala, Sweden) following the manufacturer’s instructions. All chemicals were obtained by Sigma-Aldrich (Sigma-Aldrich, St. Louis, MO).

### Conjugation of linear O-β-GlcNAc on the phosphatidylethanolamine lipid

Three forms of linear *O*-β-GlcNAc oligomers including *N-*acetylchitotriose, *N*-acetylchitotetraose and *N*-acetyl-chitopentaose were commercially available (Funakoshi, Tokyo, Japan) and chemically linked to phosphatidylethanolamine (PE) (Sigma, St. Louis, MO). In brief, glycosyl residuals were dissolved in the warmed distilled water at a concentration of 50 mg/ml (2 h, 60 °C). The PE glycolipid cores were resuspended at a concentration of 5 mg/ml in 1:2 chloroform:methanol (v/v). Sodium cyanoborohydride (NaBH_3_CN) was added and incubated overnight at 60 ºC. The successful conjugation of carbohydrate to a PE lipid was proved by a thin layer chromatography (TLC) (Merck, Darmstadt, Germany) in ethylacetate/pyridine/acetic acid/DW at the ratio of 5:5:1:3 (v/v/v/v) solvent separation system. Resolved lipids were sprayed with 1% orcinol solution and dried with heat to detect the purple spots of glycolipids.

### Recognition of O-β-GlcNAc oligomers by a CTD110.6 antibody

It has been reported that a CTD110.6 antibody is used to detect *O*-β-GlcNAc monomer on its substrate^19^. We thus used this CTD110.6 antibody to verify its recognition towards the oligomeric (tri-, tetra- and penta-) *O*-β-GlcNAc linked PE lipid using an ELISA assay. Approximately 5 μg/ml of either the PE-linked chitotriose or chitotetraose or chitopentaose mixture was added into 96-well plate and incubated (37 °C, 2 h). After extensive washes with PBS, the carbohydrates were blocked with 1% BSA in PBS, washed and incubated with 0.4 μg/ml (1:500) of a CTD110.6 antibody (Santa Cruz Biotechnology, Santa Cruz, CA) (37 °C, 2 h), followed by HRP conjugated goat anti-mouse IgG at a dilution 1:1,000 (Abcam, Cambridge, UK). Binding of antibody was detected by *O*-phenylenediamine substrate in 0.1 M Tris–HCl pH 6.8 containing 3% H_2_O_2_ (Merck-Millipore, Darmstadt, Germany). The reaction was stopped by adding 2 N H_2_SO_4_ and the developing color was quantified at 490 nm using a VERSAmax microplate reader (Molecular Devices, Sunnyvale, CA).

### Detection of naturally anchored O-β-GlcNAc polymers

Approximately 10 µg of purified *pm*TSP-II proteins were dissolved in either native loading buffer or SDS-PAGE loading buffer. Each sample was then electrophoresed on 12.5% polyacrylamide under both non-reducing and reducing conditions. The gels were then stained by FASTsilver™ Gel staining kit (Merck, Darmstadt, Germany) or Coomassie blue staining (Merck, Darmstadt, Germany). For Western blotting, both native and reducing gels were electro- transferred onto a PVDF membrane (Merck-Millipore, Darmstadt, Germany). The membrane was treated with a blocking solution (1% BSA in PBS containing 0.1% tween) for 1 h at room temperature and then incubated with 0.4 μg/ml (1:500) of a CTD110.6 monoclonal antibody overnight at 4 °C. After an extensive wash with PBST, the membrane was further incubated with 1:2,500 horse radish peroxidase (HRP) conjugated goat anti-mouse IgG (Abcam, Cambridge, UK) (room temperature, 2 h). The antigen–antibody complex was visualized by enhanced chemiluminescent method using ECL detection kit (Merck, Darmstadt, Germany). The resolved proteins on the PVDF membrane were exposed to the anti-TSP antibody^16^ and the corresponding HRP-conjugated secondary antibody in the same conditions described above.

### Immunolocalization of O-β-GlcNAc polymers in shrimp ovarian tissues

Mature ovarian were fixed with Davidson’s fixative overnight, then embedded in paraffin solution. The paraffin sections of mature ovary (5-μm thick) were dewaxed with xylene, rehydrated with a decreasing grading alcohol, then treated with 1% hydrogen peroxide (H_2_O_2_) to quench any endogenous peroxidase activity. Subsequently, the sections were incubated with a blocking solution (2% BSA in PBS containing 0.4% Triton-X) for 30 min at room temperature, followed by 2 μg/ml (1:100) dilution of CTD110.6 antibody at 4 °C overnight. Thereafter, the sections were treated with 1:500 goat anti-mouse IgG conjugated with HRP (Abcam, Cambridge, UK) for 2 h at room temperature. The enzymatic product was visualized by a Nova Red peroxidase substrate kit (Vector laboratories, Burlingame, CA). Sections were counterstained with 0.1% hematoxylin and photographed using a DM3000 Leica microscopy.

### Chitinase digestion and testing of the remaining glycoconjugates on pmTSP-II

The function of naturally conjugated *O-*β-GlcNAc in TSP chain association was tested by a chitinase digestion experiment. An equal volume of the purified *pm*TSP-II (1 mg/ml) was subjected to 1, 0.5, 0.25 and 0.1 units (U) chitinase digestion (Sigma Aldrich, specific activity is 200 U/mg) at the various time points (3 and 24 h) with a gentle agitation. The reactions were stopped by placing the mixtures on ice (4 °C). Alternatively, the purified *pm*TSP-II proteins were also treated with deglycosylation agents, trifluoromethanesulfonic (TFMSA)^37^ and sodium metaperiodate (NaIO_4_) (Sigma-Aldrich), as well as strong denaturizing agents, 7 M urea in 0.1 N HCl (Sigma-Aldrich) and 6 × loading buffer. The samples at various enzyme concentrations and digestion conditions were then resolved by gel electrophoresis either under a 5–20% continuous gradient native condition or SDS-PAGE, followed by silver staining. These digested *pm*TSP-II proteins were transferred onto PVDF membrane and probed with a CTD110.6 antibody to visualize the remaining *O-*β-GlcNAc residues on *pm*TSP-II protein under the antibody probing conditions described above.

### Ethical approval

All animals procedures were conducted in accordance with the ethical standards of the guideline of the Animal Care Committee, Mahidol University (MUSC-IACUC, protocol no. 2016/014).

## Supplementary Information

Supplementary Figure 3.

Supplementary Figure 3.

Supplementary Figure 4.

Supplementary Legends.

## Data Availability

The datasets analyzed during the current study are available in the National center for Biotechnology information (NCBI) (https://www.ncbi.nlm.nih.gov) repository.
