# Supplementary figures and images for: Shrimp thrombospondin (TSP): presence of O-β1,4 N-acetylglucosamine polymers and its function in TSP chain association in egg extracellular matrix
Source: Sci Rep. 2022 May 13;12:7925. doi: 10.1038/s41598-022-11873-7 (PMC9106747; doi:10.1038/s41598-022-11873-7)

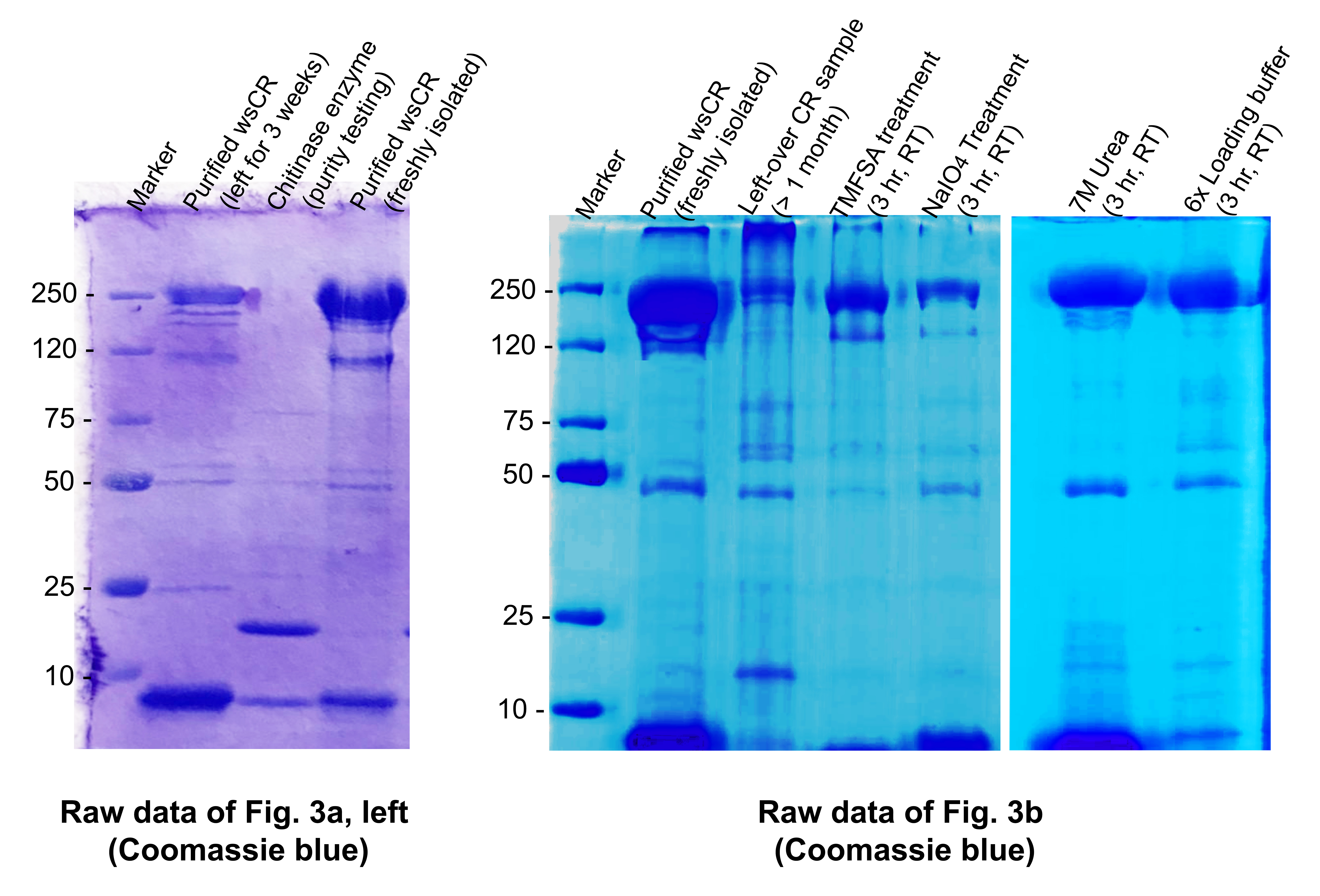

Supplement: Supplementary file 1 — Supplementary Figure 3. [file 41598_2022_11873_MOESM1_ESM.jpg]

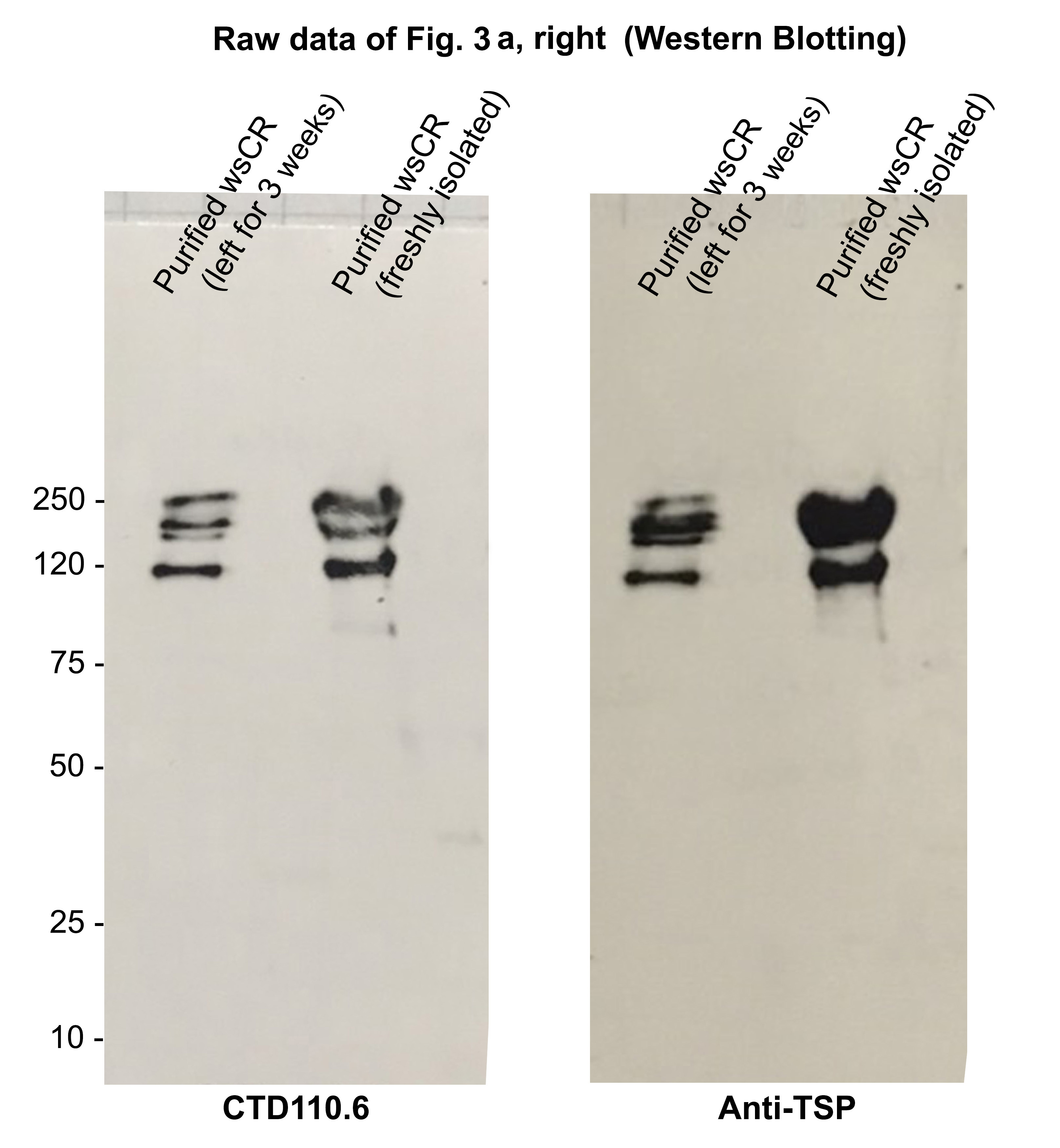

Supplement: Supplementary file 2 — Supplementary Figure 3. [file 41598_2022_11873_MOESM2_ESM.jpg]

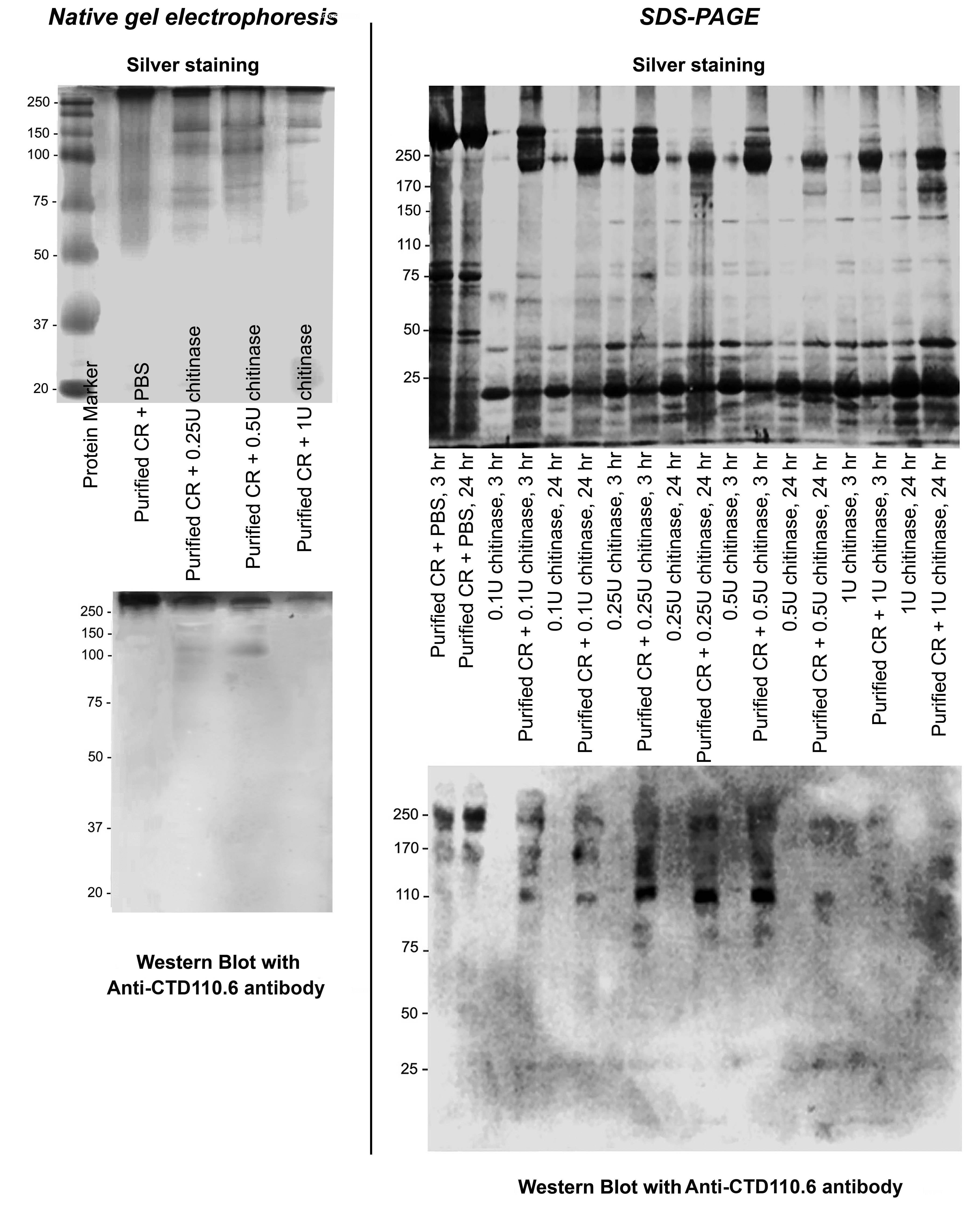

Supplement: Supplementary file 3 — Supplementary Figure 4. [file 41598_2022_11873_MOESM3_ESM.jpg]
