# Supplementary material for: Shrimp thrombospondin (TSP): presence of O-β1,4 N-acetylglucosamine polymers and its function in TSP chain association in egg extracellular matrix
Source: Sci Rep. 2022 May 13;12:7925. doi: 10.1038/s41598-022-11873-7 (PMC9106747; doi:10.1038/s41598-022-11873-7)
Supplement: Supplementary file 4 — Supplementary Legends. [file 41598_2022_11873_MOESM4_ESM.docx]

**Raw data of Figure 3a (left panel) and Figure 3b** (Coomassie blue staining of SDS-PAGE).

**Raw data of Figure 3a** **(right panel)** (Western Blotting with CTD 110.6 (left) and anti-TSP antibodies (right)).

**Raw data of Figure 4** A native gradient gel electrophoresis (left panel) and SDS-PAGE (right panel) were either stained with silver staining (upper panels) or a CTD110.6 antibody (lower panels).
